# Detection of non-coding RNAs on the basis of predicted secondary structure formation free energy change

**DOI:** 10.1186/1471-2105-7-173

**Published:** 2006-03-27

**Authors:** Andrew V Uzilov, Joshua M Keegan, David H Mathews

**Affiliations:** 1 Department of Biochemistry & Biophysics, University of Rochester Medical Center, 601 Elmwood Avenue, Box 712, Rochester, New York 14642, USA; 2 Department of Biostatistics & Computational Biology, University of Rochester Medical Center, 601 Elmwood Avenue, Box 712, Rochester, New York 14642, USA; 3 Center for Pediatric Biomedical Research, University of Rochester Medical Center, 601 Elmwood Avenue, Box 712, Rochester, New York 14642, USA

## Abstract

**Background:**

Non-coding RNAs (ncRNAs) have a multitude of roles in the cell, many of which remain to be discovered. However, it is difficult to detect novel ncRNAs in biochemical screens. To advance biological knowledge, computational methods that can accurately detect ncRNAs in sequenced genomes are therefore desirable. The increasing number of genomic sequences provides a rich dataset for computational comparative sequence analysis and detection of novel ncRNAs.

**Results:**

Here, Dynalign, a program for predicting secondary structures common to two RNA sequences on the basis of minimizing folding free energy change, is utilized as a computational ncRNA detection tool. The Dynalign-computed optimal total free energy change, which scores the structural alignment and the free energy change of folding into a common structure for two RNA sequences, is shown to be an effective measure for distinguishing ncRNA from randomized sequences. To make the classification as a ncRNA, the total free energy change of an input sequence pair can either be compared with the total free energy changes of a set of control sequence pairs, or be used in combination with sequence length and nucleotide frequencies as input to a classification support vector machine. The latter method is much faster, but slightly less sensitive at a given specificity. Additionally, the classification support vector machine method is shown to be sensitive and specific on genomic ncRNA screens of two different *Escherichia coli *and *Salmonella typhi *genome alignments, in which many ncRNAs are known. The Dynalign computational experiments are also compared with two other ncRNA detection programs, RNAz and QRNA.

**Conclusion:**

The Dynalign-based support vector machine method is more sensitive for known ncRNAs in the test genomic screens than RNAz and QRNA. Additionally, both Dynalign-based methods are more sensitive than RNAz and QRNA at low sequence pair identities. Dynalign can be used as a comparable or more accurate tool than RNAz or QRNA in genomic screens, especially for low-identity regions. Dynalign provides a method for discovering ncRNAs in sequenced genomes that other methods may not identify. Significant improvements in Dynalign runtime have also been achieved.

## Background

RNA plays many important biological roles other than as a transient carrier of amino acid sequence information. It catalyzes peptide bond formation [1,2], participates in protein localization [3], serves in immunity [4], catalyzes intron splicing and RNA degradation [5], serves in dosage compensation [6], is an essential subunit in telomerase [7], guides RNA modification [8,9], controls development [10,11], and has an abundance of other regulatory functions [12-14].

Non-coding RNAs (ncRNAs) are transcripts that have function without being translated to protein. The number of known ncRNAs is growing quickly [15-17], and their significance had been severely underestimated in classic models of cellular processes [18]. It is desirable to develop high-throughput methods for discovery of novel ncRNAs for greater biological understanding and for discovering candidate drug targets.

However, novel ncRNAs are difficult to detect in conventional biochemical screens [19]: they are frequently short [18,20], often not polyadenylated [19], and might only be expressed under specific cellular conditions [20-22]. Experimental screens have found many ncRNAs [23,24], but have demonstrated that no single screen is capable of discovering all known ncRNAs for an organism. A more effective approach, demonstrated in previous studies [25-30], may be to first detect ncRNA candidates computationally, then verify them biochemically. Considering the number of available whole genome sequences [31-37], this approach can be applied to a large and diverse dataset, and has massive potential for novel ncRNA discovery.

The effectiveness of a computational ncRNA detection/classification method is determined by measuring its sensitivity and specificity on a test set of known ncRNAs and negative sequences. Sensitivity and specificity are defined as:

sensitivity=true positivestrue positives+false negatives     [eq. 1]
 MathType@MTEF@5@5@+=feaafiart1ev1aaatCvAUfKttLearuWrP9MDH5MBPbIqV92AaeXatLxBI9gBaebbnrfifHhDYfgasaacH8akY=wiFfYdH8Gipec8Eeeu0xXdbba9frFj0=OqFfea0dXdd9vqai=hGuQ8kuc9pgc9s8qqaq=dirpe0xb9q8qiLsFr0=vr0=vr0dc8meaabaqaciaacaGaaeqabaqabeGadaaakeaacqqGZbWCcqqGLbqzcqqGUbGBcqqGZbWCcqqGPbqAcqqG0baDcqqGPbqAcqqG2bGDcqqGPbqAcqqG0baDcqqG5bqEcqGH9aqpdaWcaaqaaiabbsha0jabbkhaYjabbwha1jabbwgaLjabbccaGiabbchaWjabb+gaVjabbohaZjabbMgaPjabbsha0jabbMgaPjabbAha2jabbwgaLjabbohaZbqaaiabbsha0jabbkhaYjabbwha1jabbwgaLjabbccaGiabbchaWjabb+gaVjabbohaZjabbMgaPjabbsha0jabbMgaPjabbAha2jabbwgaLjabbohaZjabgUcaRiabbAgaMjabbggaHjabbYgaSjabbohaZjabbwgaLjabbccaGiabb6gaUjabbwgaLjabbEgaNjabbggaHjabbsha0jabbMgaPjabbAha2jabbwgaLjabbohaZbaacaWLjaGaaCzcaiabcUfaBjabbwgaLjabbghaXjabc6caUiabbccaGiabigdaXiabc2faDbaa@80C7@

specificity=true negativestrue negatives+false positives     [eq. 2]
 MathType@MTEF@5@5@+=feaafiart1ev1aaatCvAUfKttLearuWrP9MDH5MBPbIqV92AaeXatLxBI9gBaebbnrfifHhDYfgasaacH8akY=wiFfYdH8Gipec8Eeeu0xXdbba9frFj0=OqFfea0dXdd9vqai=hGuQ8kuc9pgc9s8qqaq=dirpe0xb9q8qiLsFr0=vr0=vr0dc8meaabaqaciaacaGaaeqabaqabeGadaaakeaacqqGZbWCcqqGWbaCcqqGLbqzcqqGJbWycqqGPbqAcqqGMbGzcqqGPbqAcqqGJbWycqqGPbqAcqqG0baDcqqG5bqEcqGH9aqpdaWcaaqaaiabbsha0jabbkhaYjabbwha1jabbwgaLjabbccaGiabb6gaUjabbwgaLjabbEgaNjabbggaHjabbsha0jabbMgaPjabbAha2jabbwgaLjabbohaZbqaaiabbsha0jabbkhaYjabbwha1jabbwgaLjabbccaGiabb6gaUjabbwgaLjabbEgaNjabbggaHjabbsha0jabbMgaPjabbAha2jabbwgaLjabbohaZjabgUcaRiabbAgaMjabbggaHjabbYgaSjabbohaZjabbwgaLjabbccaGiabbchaWjabb+gaVjabbohaZjabbMgaPjabbsha0jabbMgaPjabbAha2jabbwgaLjabbohaZbaacaWLjaGaaCzcaiabcUfaBjabbwgaLjabbghaXjabc6caUiabbccaGiabbkdaYiabc2faDbaa@8024@

where true positives are ncRNAs that are detected by the method, true negatives are sequences that are not ncRNA and are not classified as ncRNA by the method, false positives are sequences that are not ncRNA, but are classified as ncRNA by the method, and false negatives are ncRNAs that are missed by the method.

Generally, there is a tradeoff between sensitivity and specificity – tailoring a computational method to increase one measurement may decrease the other. Throughout this paper, receiver operating characteristic (ROC) curves are used to visually express the quality of a ncRNA classification method by plotting sensitivity as a function of the false positive rate (1 – specificity), providing a complete description of all possible sensitivity/specificity tradeoffs. It should be noted that in a whole genome screen, high specificity is more essential than high sensitivity due to the large ratio of non-ncRNA sequence to ncRNA sequence. Low specificity results in an overwhelming number of false positives, swamping the number of true positives, and increasing the difficulty, time, and cost of a biochemical verification screen.

It has been proposed that ncRNAs may form secondary structures that are more stable than would be expected from non-ncRNA sequences of the same nucleotide or dinucleotide composition [38-41]. This hypothesis has been controversial; it has been suggested that it is not true, or at least that the stability difference is not statistically significant enough to be a sensitive and specific criterion for classifying sequences as ncRNA [19] (also claimed on the basis of a small set of tRNA in [42]). However, the program RNAz was recently reported [43] to use folding free energy changes of single sequences, combined with a structure conservation index (SCI) determined from a fixed, multiple sequence alignment, to effectively detect ncRNA. The SCI is the ratio of the consensus secondary structure free energy change (which also includes terms rewarding mutations evidencing structure conservation) determined by RNAalifold [44] to the average folding free energy change for each sequence determined alone. This indicates that incorporating secondary structure conservation into a model based on folding free energy change improves the quality of prediction.

Here, the effectiveness of the program Dynalign [45,46] as a tool for detection of ncRNA on the basis of predicted folding free energy change is investigated. Dynalign is a dynamic programming algorithm for simultaneously computing the lowest free energy common secondary structure and the structural alignment for two sequences. In brief, Dynalign minimizes ΔG_total_:

ΔG°_total _= ΔG°_1 _+ ΔG°_2 _+ (number of gaps in alignment) × ΔG°_gap penalty _   [eq. 3]

where ΔG°_1 _and ΔG°_2 _are the predicted folding free energy changes of secondary structures of sequence 1 and sequence 2, respectively, and ΔG°_gap penalty _is a penalty applied for each gap in the alignment. Only conserved helices, i.e. those that appear in both sequences, are predicted. The conformational free energy changes are predicted using an empirical nearest neighbor model [47-49] and ΔG°_gap penalty _was empirically determined by maximizing structure prediction accuracy [45]. Dynalign predicts secondary structure with significantly greater accuracy than single sequence structure prediction methods because of the additional information contained in the structural alignment [45,46]. It requires no sequence identity between the two sequences to perform well because there are no energy terms (equation 3) that address sequence identity. Therefore, Dynalign is robust for cases in which extensive covariation of base-paired nucleotides exists as a result of sequence evolution.

Dynalign is initially implemented in this paper as a computational ncRNA classifier by using it to compute the ΔG°_total _of an input sequence pair, then comparing that value to the mean of ΔG°_total_s of control sequence pairs generated specifically for that input pair. If the ΔG°_total _of the input sequence pair is sufficiently lower than the mean ΔG°_total _of the set of controls, the input sequences are classified as ncRNA. The *z *score is used to quantify this difference, defined as:

*z *= (*x *- μ)/σ     [eq. 4]

where *x *is the ΔG°_total _of the input sequence pair, and μ and σ are the mean and standard deviation of the ΔG°_total_s of sequence pairs in the control set, respectively. Therefore, the *z *score is just the number of standard deviations that the ΔG°_total _of the input sequence pair is above or below the mean of its set of controls.

It should be noted that the definition of *z *score implies that the control set values follow a normal distribution, but it has been noted that the distribution of ΔG°s for single sequences is actually extreme value with skew towards lower folding free energies [19]. Tests (data not shown) suggest that the distributions of ΔG°_total_s of sequence pairs in control sets are also skewed towards lower free energies. However, the *z *score is an effective measure for classification and has been used in this manner elsewhere [19,41,43,44].

This approach is tested on a large database of known 5S rRNA and tRNA sequences and artificially generated negatives, demonstrating that the *z *score based on the ΔG°_total _can be used as a sensitive and specific classification measure. These results are also compared to RNAstructure [49], a dynamic programming algorithm for single sequence secondary structure prediction by free energy minimization. Also, a support vector machine (SVM) is implemented to speed the classification process by training an SVM classifier that does not require a control set for an input sequence pair.

Additionally, the capability to use Dynalign as an effective genomic ncRNA screening tool is illustrated with a whole genome screen on a crude alignment of the *Escherichia coli *and *Salmonella typhi *genomes [31,32], which contain a significant number of known ncRNAs. Many methods have been employed for genomic screens for ncRNAs of specific families [50-58]; benchmarks and discussion in this paper are focused on the premise of using Dynalign as a general genomic screening tool for diverse, novel ncRNAs.

The above tests are benchmarked against two leading ncRNA prediction programs, QRNA [59] (version 2.0.2c) and RNAz [43] (version 0.1.1). RNAz uses a regression SVM to compute a *z *score for each sequence in a multiple sequence alignment, then uses the mean of those *z *scores and the SCI as input to a classification SVM. While structure predictions by Dynalign and RNAz are based on calculating the most stable secondary structure using experimentally determined thermodynamic parameters, QRNA uses a fully probabilistic covariance analysis approach that compares scores of three models – ncRNA, open reading frame, or other (null hypothesis) – for a pair of sequences.

Unlike Dynalign, which optimizes its own structural alignment, both QRNA and RNAz require a fixed sequence alignment as input. It is shown here that at low pairwise sequence identity, the Dynalign approach outperforms the fixed alignment approach. Additionally, Dynalign is shown to be a more sensitive ncRNA finder on whole genome screen tests.

## Results and discussion

### Improving time and memory performance of Dynalign

Dynalign's complexity is O(*N*^3^*M*^3^) in time and O(*N*^2^*M*^2^) in storage, where *N *is the length of the shorter sequence and *M *is the maximum separation parameter that limits the set of sequence alignments that are considered [45,46]. For nucleotide *i *in the first sequence to align to nucleotide *k *in the second sequence:

| *i *- *k *| = *M *    [eq. 5]

must be satisfied. The *M *parameter therefore reduces the set of alignments that are considered by Dynalign and hence the computational cost. Similar constraints have been used by others [60-62] to provide computational tractability.

To improve the efficiency of Dynalign, two strategies have been employed. The first was to recast the implementation of the *M *parameter to a form that scales with the difference in sequence length of the two sequences, so that for *i *and *k *to align:

| *i *× (*N*_2_/*N*_1_) - *k *| = *M *    [eq. 6]

must be satisfied, where *N*_1 _is the total length of the first sequence and *N*_2 _is the total length of the second sequence. This constraint automatically allows the 3' ends of the sequences (*i *= *N*_1 _and *k *= *N*_2_) to align for any *M *and any difference in sequence length. With equation 5, *M *had to be at least as large as the difference in lengths of the sequences in order for the 3' ends of the sequences to align. Now, with equation 6, significantly smaller *M *sizes can be used with Dynalign. For example, tRNA sequences can now be folded with an *M *= 6, where previously *M *= 15 was used, resulting in a significant runtime improvement without affecting accuracy.

The second approach employed to accelerate Dynalign was to determine base pairs that are unlikely to form on the basis of single sequence folding and then not consider those pairs in the Dynalign calculation. Pairs that would result in secondary structures with free energy greater than the lowest free energy structure by more than 30%, as determined by energy dot plots [63], are excluded from consideration in the Dynalign calculation [49]. Table 1 shows that nearly 99% of known base pairs are found within this energy increment, hence this heuristic has little effect on the accuracy of Dynalign calculations. This pre-computation of structural information by single sequence secondary structure prediction is similar to approaches used by Hofacker *et al *[61] and Holmes [60] to speed the alignment of RNA sequences using secondary structure information.

For the benchmarks performed previously [46], using these two methods does not lower the accuracy of Dynalign secondary structure predictions (Table 2). Table 3 shows the calculation time and memory requirements for three pairs of RNA sequences with *N *from 77 to 217 before and after both of the above improvements. Calculations that use the improved Dynalign are completed in less than a twentieth of the time required for calculations using the previous Dynalign. The calculation time is now reduced to a level similar to FOLDALIGN [62], another dynamic programming algorithm that determines the secondary structure common to two unaligned sequences. The revised Dynalign is available for download from the Mathews lab website [64] as both source code for local compilation and as part of the RNAstructure package for Microsoft Windows.

### Tests by *z *score classification of single sequences

To test the effectiveness of classifying single sequences as ncRNA on the basis of a folding free energy change, RNAstructure [49] was used to compute the minimum folding free energy change for a test set of 1,582 known 5S rRNA, tRNA, and negative sequences. A negative sequence was generated from each real ncRNA by the Altschul-Erikson sequence shuffle that exactly preserves the nucleotide and dinucleotide (i.e. AA, AU, AC, etc.) frequencies of the real ncRNA [41,65]. Because the stabilities of base pairs are predicted using a nearest neighbor model that considers the sequence identity of two stacked pairs, negative and control sequences must preserve the dinucleotide frequencies of the original sequence, while also breaking the nested base pair structure [41,42]. The negatives are needed to test the rate of false positive classification to determine specificity.

**Figure 1:**
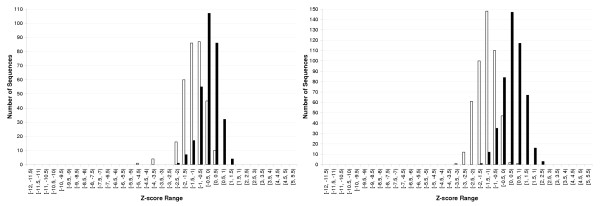
**Distribution of single sequence *z *scores for 5S rRNA, tRNA, and negative sequences**. Distributions of RNAstructure-predicted *z *scores computed on the basis of folding single sequences for 5S rRNA and negatives generated from them (left figure) and tRNA and negatives generated from them (right figure). Real ncRNA are white, negatives are black. Controls were generated by the Altschul-Erikson dinucleotide shuffle of original sequence, with 100 controls for each test set sequence. 309 5S rRNA sequences and 482 tRNA sequences, plus one negative sequence generated from each real sequence by the Altschul-Erikson shuffle, were used for the test set.

To compute the *z *score, each sequence in the test set had a control set of 100 sequences generated specifically for it using the Altschul-Erikson shuffle, and their minimum folding free energy changes were determined by RNAstructure. The *z *score histograms for 5S rRNA, tRNA, and negative sequences generated from them are shown in Figure 1.

**Figure 2:**
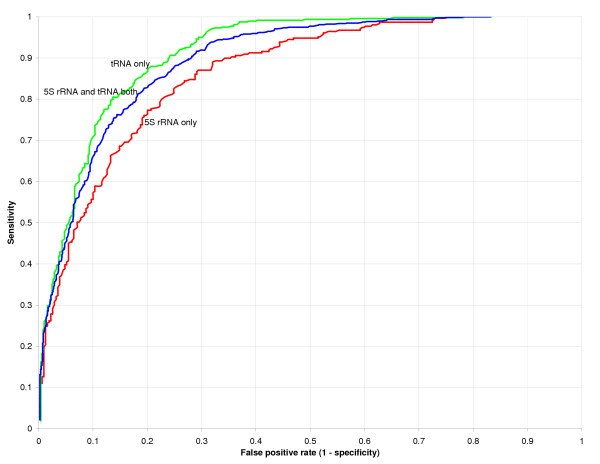
**Quality of classification using the *z *score method for single sequences**. ROC curves showing quality of classification based on single sequences, using RNAstructure-predicted *z *scores for folding free energy change. The ncRNA sequences and controls are the same as in Figure 1. Red and green show results for 5S rRNA and tRNA, respectively, when tested separately; blue shows results when both are combined into a single test set.

Sequences below a cutoff *z *score are classified as ncRNA. However, rather than pick a single *z *score cutoff for classification and report those results, iterations were performed over a wide range of *z *score cutoffs in order to construct an ROC curve (Figure 2) expressing sensitivity as a function of the false positive rate, thus showing the overall quality of the ncRNA classification method for all sensitivity/specificity tradeoffs. Figure 2 shows that tRNA sequences are classified with better sensitivity (for all specificities) than 5S rRNA sequences using either method, which suggests that tRNA sequences have a lower predicted folding free energy than 5S rRNA sequences versus matched controls.

### Tests by *z *score classification of sequence pairs

To test the effectiveness of classifying pairs of sequences as ncRNA using Dynalign on the basis of the ΔG°_total_-based *z *score, *z *scores were determined for a test set of 3,302 known 5S rRNA, tRNA, and negative sequence pairs. Negative sequence pairs were generated from real sequence pairs by shuffling the columns in the real sequence pair gapped global alignment, then removing gaps. Three control generation methods were used for each sequence pair to randomize the nucleotide order and remove the nested base pair structure while preserving other sequence properties. Because Dynalign's computation time is greater than that of RNAstructure, the number of controls per sequence pair was limited to 20 to make the calculation time feasible.

The first two control generation methods focus on preserving dinucleotide frequencies (i.e. frequencies of AA, AU, AC, etc.) and are applied to each sequence in the original pair separately, without regard for alignment. As with prediction from single sequences, because stability contributions of each base pair are dependent on the base pairs on which it is stacked, it may be necessary to control for the dinucleotide frequencies [41,42]. The first-order Markov chain sampling method for control sequence generation *approximately *preserves the original dinucleotide frequencies [41] (resulting in more variation in the control set), while the Altschul-Erikson shuffle method for control generation *exactly *preserves both nucleotide and dinucleotide frequencies, with the restriction that the first and last nucleotide of the shuffled sequence are exactly the same as the original [65].

The third control generation method is a columnwise shuffle of a global alignment that approximately preserves the percent identity of the original sequence alignment. Although removing gaps and re-aligning the columnwise shuffled sequences results in a different alignment, the change in percent identity from the original sequence pair is not as drastic as with the other two control methods. For example, columnwise shuffling a sequence pair alignment, followed by re-alignment, results in a mean percent identity change of 2.57, with a standard deviation of 2.74; however, the mean and standard deviation of percent identity change if Altschul-Erikson shuffles are used are -11.30 and 9.28, respectively. It is reasonable that randomizing sequences separately and re-aligning them results in a greater change (in most cases, a decrease) in percent identity of the sequence pair, compared to shuffling the alignment in columns.

**Figure 3:**
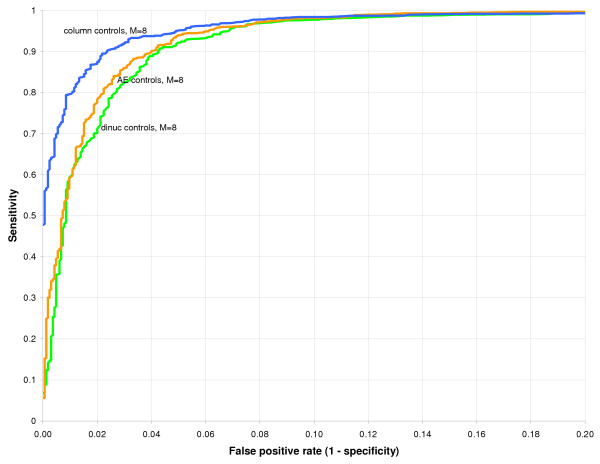
**Comparison of three methods for generating 20 controls from each input sequence pair**. ROC curves comparing three methods for generating a set of 20 controls from an input sequence pair to determine the *z *score for ncRNA classification using the Dynalign-computed ΔG°_total_. The test set contains 755 5S rRNA and 896 tRNA sequence pairs, plus one negative sequence pair generated from each real sequence pair, yielding 3,302 trial pairs total. All tests are run with the parameter *M *= 8. "dinuc controls" (green): controls are generated by sampling from a first-order Markov chain, approximately preserving dinucleotide frequencies of each original sequence. "AE controls" (orange): controls are generated by the Altschul-Erikson dinucleotide shuffle, exactly preserving dinucleotide frequencies of each original sequence. "column controls" (blue): controls are generated by a columnwise shuffle of a global sequence alignment, without regard for gap placement or local conservation.

**Figure 4:**
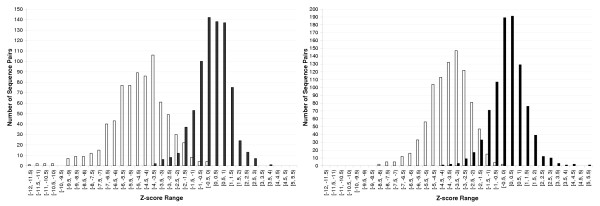
**Distribution of sequence pair *z *scores for 5S rRNA, tRNA, and negative sequences**. Distribution of *z *scores computed using the Dynalign ΔG°_total _and the columnwise shuffle control method (*M *= 8) for 5S rRNA sequence pairs and negatives generated from them (left figure) and tRNA sequence pairs and negatives generated from them (right figure). Real ncRNA are white, negatives are black. Test set is the same as for Figure 3.

ROC curves comparing effectiveness of the three control generation methods are plotted in Figure 3. The columnwise shuffle control method produces the highest sensitivities for all specificities, and is therefore the best approach of the three. The distributions of *z *scores for 5S rRNA, tRNA, and negative sequence pairs for trials using this control method are shown in Figure 4, and the separation of real ncRNA and negative sequences is significantly more distinct than in Figure 1 (single sequence *z *scores). Additionally, the Altschul-Erikson shuffle control generation method is more effective than the first-order Markov chain sampling method.

**Figure 5:**
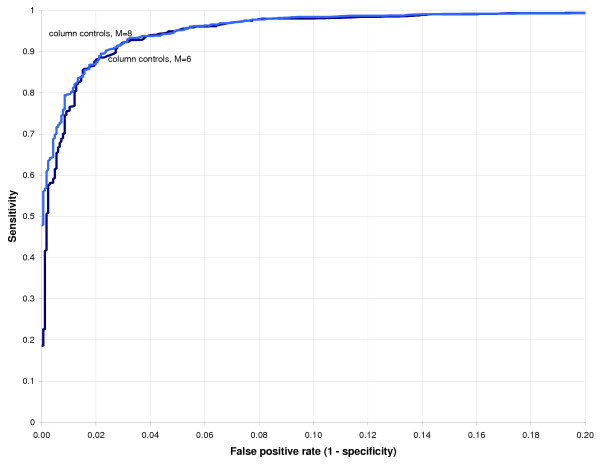
**Higher *M *parameter improves quality of classification when using the *z *score method**. ROC curves comparing effectiveness of the best control generation method for sequence pairs (i.e. columnwise shuffle of a global sequence alignment) at parameters *M *= 6 (dark blue) and *M *= 8 (light blue). Test set is same as for Figure 3. For all other control generation methods, increasing the *M *parameter value likewise increases the quality of classification (see Additional File 1 in "Additional Files" for supporting figure).

Each control generation method was also tried using two different values of the *M *parameter, *M *= 6 and *M *= 8, for computation of the input sequence pair and the control set ΔG°_total_s (columnwise shuffle control generation method results in Figure 5, complete results for all methods in Additional File 1 in "Additional Files"). It was found that the higher *M *parameter improves the quality of classification for all control generation methods, at the expense of longer runtime.

**Figure 6:**
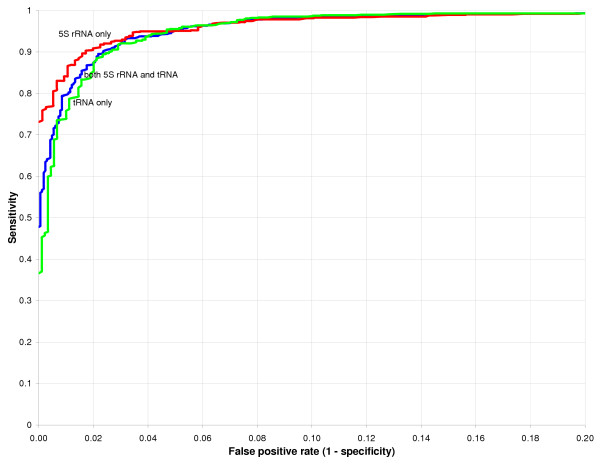
**Quality of classification using the *z *score method, broken down by ncRNA family**. ROC curves showing effectiveness of the best control generation method for sequence pairs (i.e. columnwise shuffle of global alignment at parameter *M *= 8) for 5S rRNA by itself (red), tRNA by itself (green), and both combined into one test set (blue). The 5S rRNA or tRNA sequences in the test set are the same as those used for the test set in Figures 3, 4 and 5.

**Figure 7:**
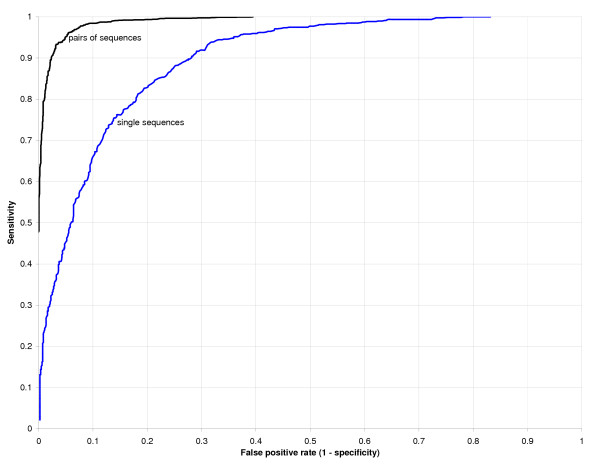
**Comparison of the *z *score classification method using single sequences versus using sequence pairs**. ROC curves comparing quality of classification based on single sequences versus based on sequence pairs, using the same free energy parameters for both. The single sequence curve (blue) is the same as in Figure 2. Black shows the best results for the sequence pair approach from Figure 3 (i.e. control generation by columnwise shuffle of global sequence alignment at parameter *M *= 8), to illustrate the difference in prediction quality.

The quality of the best control generation method is also examined when 5S rRNA and tRNA are separated into different sets and tested independently (Figure 6). It was discovered that this method is generally more sensitive at a given specificity for detecting 5S rRNA than tRNA (the opposite of the trend than observed in classification of single sequences in Figure 2). Finally, ncRNA classification using single sequences is compared with the sequence pair approach in Figure 7. This shows significantly better performance for the two sequence approach with Dynalign as compared to single sequences.

**Figure 8:**
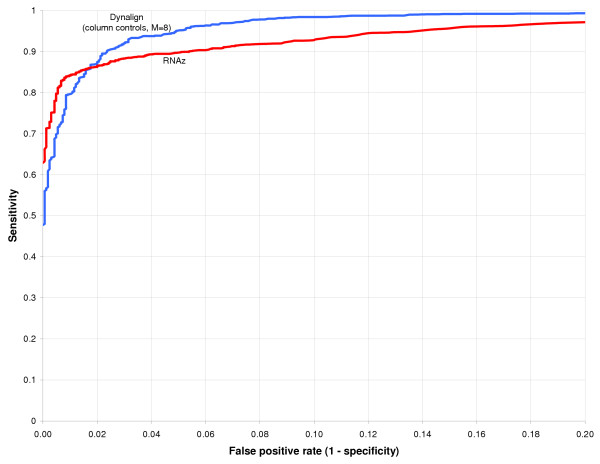
**Comparison of the Dynalign *z *score method with RNAz for sequence pairs of all identities**. ROC curves for the Dynalign *z *score classification method (running 20 controls for each input sequence pair to determine *z *score, *M *= 8; blue) and RNAz (red), both tested on the same test set of sequence pairs as in Figure 3.

Because RNAz outputs the probability (*P *value) that an input sequence alignment is ncRNA, it is possible to construct an RNAz ROC curve for the same test set as Dynalign, except by varying the sensitivity/specificity tradeoff by iterating over *P *value cutoffs for classification. If a sequence alignment input to RNAz has a *P *value greater than the cutoff, it is classified as ncRNA. The quality of classification for RNAz as compared to the Dynalign *z *score method is shown in Figure 8. While RNAz is more sensitive at specificities above approximately 98.5%, the Dynalign *z *score method is more sensitive at lower specificities.

**Figure 9:**
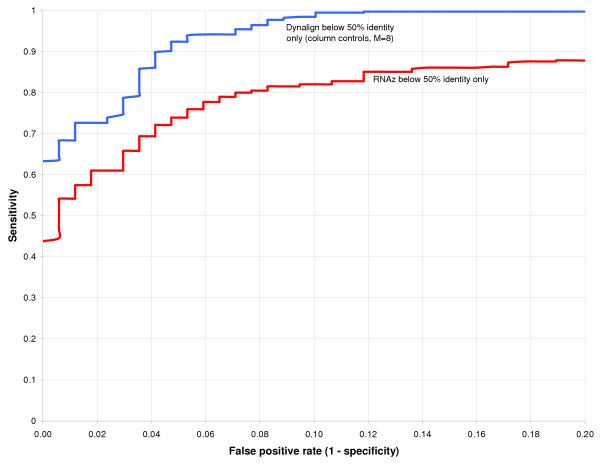
**Comparison of the Dynalign *z *score method with RNAz for sequence pairs below 50% identity**. ROC curves for the Dynalign *z *score classification method (running 20 controls for each input sequence pair, *M *= 8; blue) and RNAz (red), both tested only on those sequence pairs from the Figure 3 test set that have less than 50% sequence pair identity. Dynalign becomes more sensitive than RNAz at low sequence pair identities for all specificities.

RNAz requires pre-aligned sequences as input, which is a disadvantage at lower sequence identities because, for highly divergent sequences, an optimal *sequence *alignment prepared by an algorithm that minimizes an alignment identity score may not necessarily be the optimal *structural *alignment that takes into account the common secondary structures of the RNA sequences [66]. Dynalign does not suffer from this limitation because it simultaneously optimizes the common secondary structure and the structural alignment, and thus does not need pre-alignment of the input sequence pair. To illustrate this advantage of Dynalign over RNAz at low sequence pair identities, Figure 9 compares the ROC curves of the Dynalign *z *score method and RNAz only for sequences in the test set that are below 50% identity. At this level of low sequence identity, the Dynalign *z *score method is more sensitive than RNAz at all specificities.

### Tests by support vector machine (SVM) classification

Generating a large number of controls for each input sequence pair is an accurate, but time-consuming method for classifying sequences, making a whole genome screen costly. To speed the calculation, a support vector machine (SVM) can be used. SVMs are a set of machine learning methods capable of performing non-linear regression and classification of numerical data [67,68]. For example, RNAz uses a regression SVM to compute single sequence *z *scores and a classification SVM to determine whether a multiple sequence alignment is ncRNA or not on the basis of a set of input parameters.

To classify sequence pairs without performing explicit control calculations to generate a *z *score, a binary SVM classifier was employed (using the LIBSVM [69] implementation). The classifier takes as input the Dynalign-computed ΔG°_total _of the input sequence pair, the length of the shorter sequence, and A, U, and C nucleotide frequencies of sequence 1 and sequence 2. This Dynalign/LIBSVM classifier was trained on a set of 59,535 real and negative sequence pairs in a 1:2 ratio; the real sequence pairs were composed of two 5S rRNA or two tRNA, and two negative sequence pairs were generated from each real sequence pair using two different sequence shuffling methods. The classifier was trained to output a classification probability (*P *value) of the input sequence pair being ncRNA, thus allowing for the construction of ROC curves because the ncRNA classification cutoff could be set at any desired *P *value.

**Figure 10:**
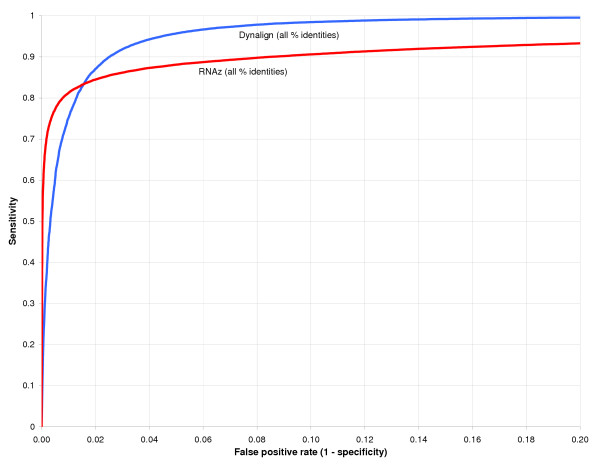
**Comparison of the Dynalign/LIBSVM classifier with RNAz for sequence pairs of all identities**. ROC curves for the Dynalign/LIBSVM classifier (blue) and RNAz (red), both based on a test set of 38,069 5S rRNA sequence pairs, 52,470 tRNA sequence pairs, plus two negative sequence pairs generated from each real sequence pair – one by a columnwise shuffle of a global alignment, one by an Altschul-Erikson dinucleotide shuffle of each sequence in the pair separately, yielding 90,539 real trial sequence pairs and 181,078 negative trial sequence pairs.

**Figure 11:**
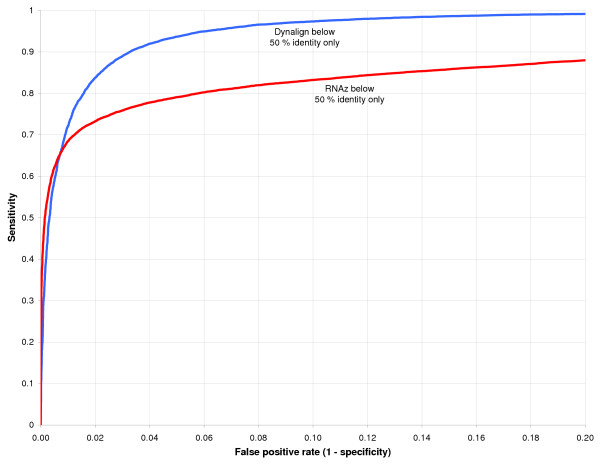
**Comparison of the Dynalign/LIBSVM classifier with RNAz for sequence pairs below 50% identity**. ROC curves for the Dynalign/LIBSVM classifier method (blue) and RNAz (red), both tested only on those sequence pairs from the Figure 10 test set that have less than 50% sequence pair identity.

To benchmark the performance of the Dynalign/LIBSVM classifier versus RNAz and QRNA, the three methods were applied to a test set of 90,539 5S rRNA and tRNA sequence pairs and 181,078 negative sequence pairs (generated in the same fashion as the set used to train the model, with two negatives for each real sequence pair). For comparison of the Dynalign/LIBSVM classifier and RNAz, ROC curves are plotted for all sequence pairs in Figure 10, and for sequence pairs below 50% identity in Figure 11. Because QRNA compares scores for three different models (ncRNA, open reading frame, or other) to make the classification, an ROC curve cannot be constructed for it as for RNAz and the Dynalign/LIBSVM method, so QRNA classification benchmark results are listed in Table 4.

The benchmarks on 5S rRNA and tRNA indicate that the Dynalign/LIBSVM classifier is more sensitive than RNAz if the desired specificity is below approximately 98.3%. However, for higher specificities, RNAz becomes more sensitive. When only sequence pairs below 50% identity are considered, the difference between the two methods in prediction quality at high specificities narrows; RNAz is more sensitive than Dynalign at above approximately 99.2% specificity, but less sensitive at all specificities below that.

Table 4 illustrates the effectiveness of the three programs broken down by percent identity of the sequence pairs. Because the Dynalign/LIBSVM classifier and RNAz allow selection of a *P *value cutoff, the cutoffs were chosen so that the specificities of the programs on the test set match those of QRNA, allowing sensitivities to be compared. It should be noted that in Table 4, the QRNA-based specificity maps to a point on the Dynalign/LIBSVM classifier and RNAz ROC curves (see Figure 10) where RNAz is more sensitive than Dynalign, which is not true for *all *specificities. Table 4 illustrates that the sensitivity of the Dynalign/LIBSVM classifier remains more consistent than RNAz or QRNA at low sequence identity. This is primarily because Dynalign optimizes the structural alignment based on secondary structure, rather than requiring a fixed alignment as input, because the optimal sequence alignment may not necessarily be the optimal structural alignment at lower identities.

**Figure 12:**
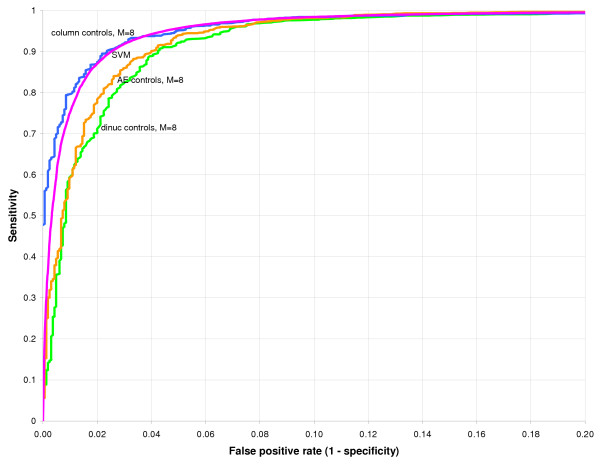
**Comparison of the Dynalign *z *score method with the Dynalign/LIBSVM classifier**. ROC curves for the Dynalign *z *score method, *M *= 8 (blue, column shuffle controls of the global alignments; orange, Altschul-Erickson dinucleotide shuffle controls; green, first-order Markov chain sampling controls) versus the Dynalign/LIBSVM classifier (pink). The *z *score ROC curves are from Figure 3; the Dynalign/LIBSVM ROC curve is from Figure 10.

While Figure 9 clearly illustrates that Dynalign is the better, albeit slower, tool for classifying low-identity sequence pairs if using the *z *score method, this apparently does not carry over as effectively into the Dynalign/LIBSVM classifier. Figure 12 illustrates the ROC curve for the Dynalign/LIBSVM classifier plotted with ROC curves for the *z *score method from Figure 3, indicating that the quality of prediction with the Dynalign/LIBSVM classifier is worse than the best *z *score control generation method, although being approximately 20 times faster because no explicit controls have to be run.

### Detection of long ncRNAs

Because the runtime complexity of Dynalign prohibits an efficient whole genome screen using long scanning windows, the hypothesis that the Dynalign/LIBSVM classifier could pick up long ncRNAs by scanning through them using short windows was tested. Three 16S rRNA and three 23S rRNA sequence pairs were chosen randomly from a database of sequences [48], and, for each pair, a global alignment was constructed. The alignments were scanned with windows of size 150 nucleotides, stepping 75 nucleotides at a time. The alignment information was removed from each window prior to input to Dynalign because Dynalign takes two unaligned sequences as input. The Dynalign/LIBSVM classifier was used to compute the probability (*P *value) of each window being ncRNA.

**Figure 13:**
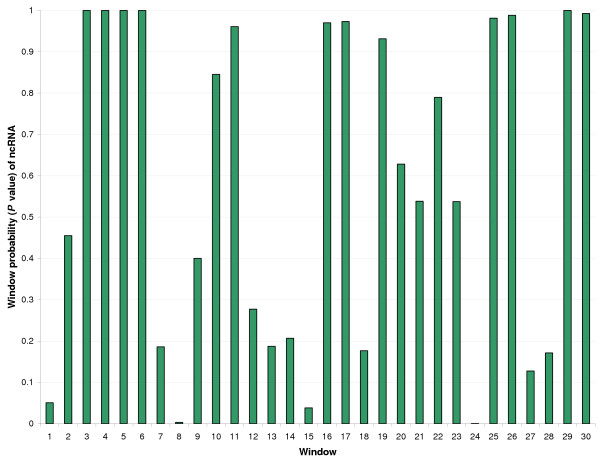
**ncRNA probabilities (*P *values) of scanning windows iterating through a 16S rRNA**. Probabilities of ncRNA computed by the Dynalign/LIBSVM classifier for 30 150-nucleotide-long scanning windows iterating through a global alignment of *Borrelia burgdorferi *and *Bacillus subtilis *16S rRNA in steps of 75.

**Figure 14:**
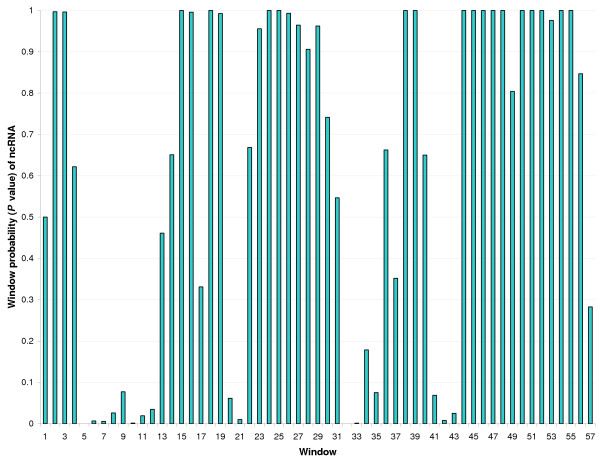
**ncRNA probabilities (*P *values) of scanning windows iterating through a 23S rRNA**. Probabilities of ncRNA computed by the Dynalign/LIBSVM classifier for 57 150-nucleotide-long scanning windows iterating through a global alignment of *Bacillus subtilis *and *Bos taurus *23S rRNA in steps of 75.

Table 5 shows the *P *values for all the windows, demonstrating that each of the long ncRNAs has at least one high-probability (*P *> 0.9) window that would detect it in a whole genome screen. In most cases the number of high-probability windows is large. This indicates that it should be possible to discover long ncRNAs in a whole genome screen by going through them in short windows. In fact, given multiple short windows for most long ncRNAs, the overall sensitivity of long ncRNA discovery should be higher than for short sequences found in only one window. Examples of the distributions of *P *values by window for representative 16S and 23S rRNA are shown in Figures 13 and 14.

### Whole genome screen using the Dynalign/LIBSVM classifier

The capability of the Dynalign/LIBSVM classifier as a ncRNA detection tool was tested on whole genome alignments of *Escherichia coli *K-12 MG1655 [31] and the main chromosome of *Salmonella enterica serovar Typhi *(*Salmonella typhi*) CT18 [32]. Two different methods of preparing a whole genome alignment were used. In each case, nucleotides known to be in open reading frames (ORFs) were removed to speed the calculation. With the WuBLASTn [70] method, nucleotides in known open reading frames (ORFs) of *E. coli *(but not *S. typhi*) were dropped before the alignment and the screen; in the MUMmer [71] method, nucleotides in known ORFs in both genomes were retained for the alignment, but dropped before the screen. This has the disadvantage that ncRNA overlapping with or complementary to ORFs would be truncated or dropped before the screen, but the lack of a significant number of such ncRNAs did not render this a problem. For example, in *E. coli*, only eight known ncRNAs partially overlap coding regions and no known ncRNAs completely exist in coding regions. Out of the known 156 *E. coli *ncRNAs, the MUMmer whole genome alignment contained 129 completely (the ncRNA was entirely within an alignment block), 3 partially (the ncRNA was truncated in the alignment block), and 24 ncRNA did not show up at all in the alignment. The WuBLASTn alignment contained 148 completely, 7 partially, and 1 ncRNA did not show up at all. Therefore, the maximum number of detectable *E. coli *ncRNAs was 132 for the MUMmer alignment, and 155 for the WuBLASTn alignment.

**Figure 15:**
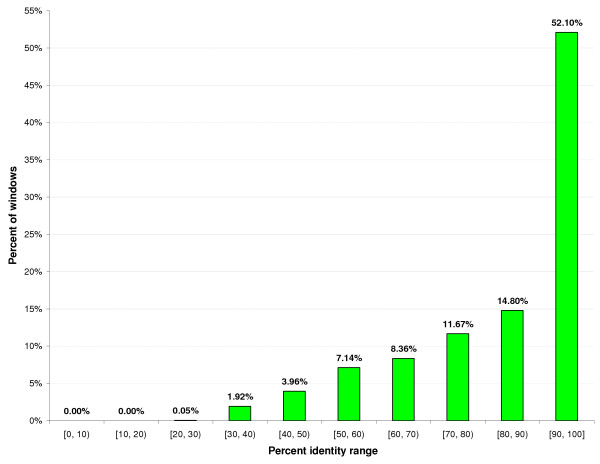
**Distribution of scanning window percent identities in the MUMmer whole genome ncRNA screen**. Histogram showing the distribution of percent identities of 15,214 genomic windows (size 150 alignment columns, scanning step size 75 alignment columns), generated from the MUMmer whole genome alignment of *E. coli *and *S. typhi*.

For the first method of preparing whole genome screen windows, a MUMmer [71] whole genome alignment was performed of the entire *E. coli *genome with the entire *S. typhi *main chromosome. Alignment columns containing known ORF nucleotides in either genome were removed after the alignment; ORF regions were retained for the alignment step only to serve as "anchors" to produce greater coverage and better align intergenic regions. The resulting alignment blocks were scanned with windows of size 150 alignment columns, stepping 75 at a time. The alignment information is removed from each window prior to input to Dynalign, but retained for input to QRNA and RNAz because they require pre-aligned sequences. This produced 15,214 total windows (counting reverse complements) containing 2,216,188 alignment columns. The distribution of percent identities for these windows is reported in Figure 15. The large number of alignment columns relative to intergenic region size is explained by the same sequences producing multiple alignment blocks, due to the quantity of repetitive elements in both genomes. After screening, overlapping and contiguous windows that are classified as ncRNA are merged and considered a single ncRNA.

Table 6 shows the results of the MUMmer whole genome screen at various *P *value cutoffs, compared against RNAz at the same cutoffs and QRNA. Given our current knowledge of ncRNAs in these genomes, the Dynalign/LIBSVM classifier is the most sensitive method for genomic screening, picking up a greater quantity of known ncRNAs. It also appears to generate either less or a roughly equivalent number of "other" hits – high-probability contiguous regions that are not annotated in the sources of known ncRNA that were used for this screen. It is currently unknown whether this indicates that the Dynalign/LIBSVM classifier method is more specific, because either fewer false positives are generated, or fewer previously unknown ncRNAs are discovered, or a combination of both. Considering the large number of these "other" hits, it is likely that this indicates a lower genomic false positive rate, but this cannot be conclusively determined. The total number of nucleotides in these "other" regions is given in Table 6 for a crude estimate of the number of probes that would be required for a biochemical verification screen.

**Figure 16:**
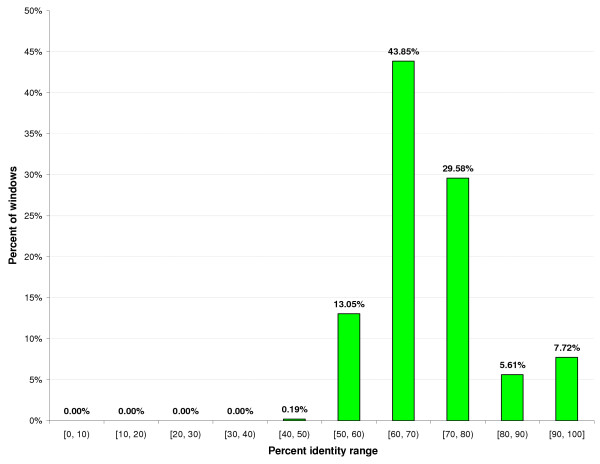
**Distribution of scanning window percent identities in the WuBLASTn whole genome ncRNA screen**. Histogram showing the distribution of percent identities of 90,404 genomic windows (size 150 alignment columns, scanning step size 75 alignment columns), generated from the WuBLASTn whole genome alignment of *E. coli *and *S. typhi*.

For the second method of preparing whole genome screen windows, intergenic regions of *E. coli *(defined as the entire genome minus known ORFs, resulting in 587,347 intergenic nucleotides) were used as WuBLASTn [70] queries against the entire *S. typhi *main chromosome, resulting in 90,404 total windows (counting the reverse complements) containing 10,265,161 alignment columns. The distribution of percent identities for these windows is reported in Figure 16. Like in the MUMmer alignment, the large number of alignment columns is due to the same sequences appearing in multiple alignment blocks. The windows were created by scanning through the resulting WuBLASTn alignment blocks in the same manner as with the MUMmer screen, using windows of size 150 alignment columns, step size 75. The alignment information was removed from each window prior to input to Dynalign, but retained for input to QRNA and RNAz because they require pre-aligned sequences. Once again, after screening, contiguous or overlapping windows classified as ncRNA were merged into single ncRNA.

The results of the WuBLASTn genomic screen listed in Table 7 differ from the results of the MUMmer genomic screen (Table 6). The number and coverage of "other" hits in *S. typhi *is much greater than in *E. coli *(whereas in the MUMmer screen they were comparable), presumably because *E. coli *intergenic regions are used as queries against the entire *S. typhi *chromosome that here, unlike in the MUMmer screen, did not have any ORFs removed prior to generating scanning windows, thus resulting in more *S. typhi *sequence present. The performance of the Dynalign/LIBSVM classifier and RNAz at the *P *> 0.99 cutoff in the WuBLASTn screen is comparable to their performance at the *P *> 0.5 cutoff in the MUMmer screen; QRNA also seems to be more sensitive and less specific in the WuBLASTn screen than MUMmer. This indicates that a MUMmer whole genome alignment would be more desirable for high-specificity whole genome screens.

The complete datasets for both genomic screens are presented in "Additional Files." Additional Files 2 and 3 give the classification of each window in the MUMmer and WuBLASTn genome screens, respectively. Additional Files 4 and 5 likewise provide the input data to the SVM classifier for the MUMmer and WuBLASTn genome screens.

## Conclusion

It has been shown that the ΔG°_total _calculated by Dynalign can be used as an effective parameter for detecting ncRNAs. Also, because Dynalign predicts a secondary structure common to two sequences, it is possible to incorporate additional structure-based parameters into the classification model. A recent benchmark of various structural alignment programs [66] reports that Dynalign structural alignments are among the best at reflecting conserved secondary structure, becoming the best at sequence identities below 50%. The potential of Dynalign as a ncRNA detection tool can be yet further explored. For example, it would be interesting to see if methods could be improved if strictly probabilistic or evolution-based scores [72] were added as input to the Dynalign/LIBSVM classifier. Additionally, considering that all testing here was based on only two ncRNA families, it would also be interesting to test how well the Dynalign/LIBSVM classifier would perform if the training set were made more diverse, or if the SVM was optimized further. The LIBSVM input data set for all possible pairwise alignments of 5S rRNA, tRNA, and negative sequences generated from them are presented in Additional File 6 in "Additional Files" for such purposes.

**Figure 17:**
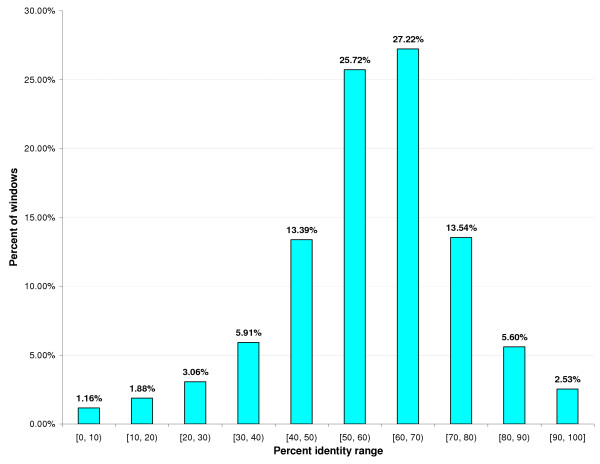
**Distribution of percent identities of 50-nucleotide windows in the human-mouse genome alignment**. The BLASTZ pairwise alignment of the human and mouse genomes [73] is broken down into 50-nucleotide-long non-overlapping windows and the percent identity for each is calculated, then plotted in this histogram. There are 22,456,315 windows total.

The advantages of using Dynalign over existing ncRNA detection methods are that it is more sensitive at most specificities and that it produces higher quality predictions at low sequence identities. The latter is important, since the number of conserved low-identity regions in some genomes of interest may be high. For example, Figure 17 illustrates a distribution of percent identities of a human-mouse BLASTZ genome alignment [73] broken down into 50 nucleotide non-overlapping windows. 25% of the alignment is in the below 50% identity region where the Dynalign *z *score method outperforms RNAz. Additionally, Table 4 seems to indicate that the Dynalign/LIBSVM classification method is more consistent across varying percent identities than the other two programs.

The disadvantages to Dynalign as a ncRNA detection method are that the number of input sequences is currently limited to two, the algorithm does not allow pseudoknots (a common limitation for secondary structure prediction algorithms), and that the runtime is longer than that of many other ncRNA classification programs, especially in the case of explicitly running controls for each input sequence pair; however, optimizations resulting in significant decreases in Dynalign runtime have been achieved as shown in Table 3. While control generation can be circumvented by using a classification SVM, the quality of prediction of such a method (as implemented and benchmarked here) appears to drop slightly. However, this simple classification SVM approach, which does not directly incorporate a *z *score into the classification model, is still more sensitive for known ncRNAs in a whole genome screen than RNAz or QRNA. It may be possible to improve the quality of classification by using a regression model to determine the *z *score separately from the classification SVM step, which is a strategy successfully employed by RNAz, except that in this case the *z *score would be based on the ΔG°_total_s of sequence pairs instead of single sequences, increasing the complexity of the regression model.

The FOLDALIGN program [62,74] is closely related to Dynalign and can also be used for ncRNA detection. FOLDALIGN also uses a dynamic programming algorithm to find the secondary structure common to two, unaligned sequences and the sequence alignment that facilitates the structure. FOLDALIGN should therefore share the same advantages and disadvantages that Dynalign has for ncRNA detection at low sequence identity. FOLDALIGN maximizes a score that includes a subset of the free energy change nearest neighbor parameters [47-49] and terms that score sequence similarity [58]. A scanning version of FOLDALIGN has been reported [62] that takes long sequences as input, but limits the length of structural motifs to a parameter, λ, and so does not require that the sequence be broken into windows.

Because it is fast, prediction from single sequences (such as using RNAstructure [48]) could be used as a rapid pre-filtering step to eliminate a large number of genomic sequence when doing a whole genome screen using these methods. For example, Figures 2 and 7 indicate that at 36% specificity, the sensitivity of prediction is approximately 99% for 5S rRNA and tRNA tests using RNAstructure. Assuming these numbers are indicative of performance on all ncRNA families, we could use single sequence prediction to quickly eliminate 36% of the negatives in a whole genome screen without sacrificing an overwhelming majority of the real ncRNA. Then, the reduced amount of sequence could be screened with the more time-consuming approach of prediction from sequence pairs, thus speeding up the overall screen.

The Dynalign ncRNA detection method that we have outlined is computationally costly, but feasible for analysis of long genomes. For example, we estimate that a Dynalign/LIBSVM screen (using 150-nucleotide-long scanning windows, step size 75) of the human-mouse whole genome alignment regions below 50% sequence identity in Figure 17, which contain approximately 563 million alignment columns (this includes the reverse complements of each window), would require approximately 1.4 CPU years after single sequence pre-filtering, or approximately 100 days of wall time on a reasonably sized 50-CPU computation cluster. Additionally, other pre-filtering methods could be employed to eliminate repetitive and other sequences prior to the Dynalign computation.

## Methods

### Local and global Dynalign implementations

The original Dynalign algorithm performs global alignments of the two sequences, i.e. gaps are penalized at the ends of the alignments by applying the ΔG°_gap penalty _term for each gap when calculating the value of ΔG°_total_. To facilitate calculations for ncRNA discovery, a local alignment option was programmed. In the local alignment Dynalign, the per nucleotide gap penalty (ΔG°_gap penalty_) is not applied to gaps at either end of either sequence in the alignment. Because the energy function (equation 3) contains no terms for sequence matching, this allows the local Dynalign to find optimal structural alignments with any portion of each sequence.

### Generation of global alignments and calculation of percent identities

To generate global alignments of two sequences, the EMBOSS (version 2.9.0) [75] Stretcher global alignment tool (with default parameters) was used. All percent identities are calculated as follows:

% identity=# of alignment columnswith matching nucleotidestotal # of alignment columns(including columns with gaps)     [eq. 7]
 MathType@MTEF@5@5@+=feaafiart1ev1aaatCvAUfKttLearuWrP9MDH5MBPbIqV92AaeXatLxBI9gBaebbnrfifHhDYfgasaacH8akY=wiFfYdH8Gipec8Eeeu0xXdbba9frFj0=OqFfea0dXdd9vqai=hGuQ8kuc9pgc9s8qqaq=dirpe0xb9q8qiLsFr0=vr0=vr0dc8meaabaqaciaacaGaaeqabaqabeGadaaakeaacqqGLaqjcqqGGaaicqqGPbqAcqqGKbazcqqGLbqzcqqGUbGBcqqG0baDcqqGPbqAcqqG0baDcqqG5bqEcqGH9aqpdaWcaaabaiqabaGaee4iamIaeeiiaaIaee4Ba8MaeeOzayMaeeiiaaIaeeyyaeMaeeiBaWMaeeyAaKMaee4zaCMaeeOBa4MaeeyBa0MaeeyzauMaeeOBa4MaeeiDaqNaeeiiaaIaee4yamMaee4Ba8MaeeiBaWMaeeyDauNaeeyBa0MaeeOBa4Maee4CamhabaGaee4DaCNaeeyAaKMaeeiDaqNaeeiAaGMaeeiiaaIaeeyBa0MaeeyyaeMaeeiDaqNaee4yamMaeeiAaGMaeeyAaKMaeeOBa4Maee4zaCMaeeiiaaIaeeOBa4MaeeyDauNaee4yamMaeeiBaWMaeeyzauMaee4Ba8MaeeiDaqNaeeyAaKMaeeizaqMaeeyzauMaee4Camhaaqaaceqaaiabbsha0jabb+gaVjabbsha0jabbggaHjabbYgaSjabbccaGiabbocaJiabbccaGiabb+gaVjabbAgaMjabbccaGiabbggaHjabbYgaSjabbMgaPjabbEgaNjabb6gaUjabb2gaTjabbwgaLjabb6gaUjabbsha0jabbccaGiabbogaJjabb+gaVjabbYgaSjabbwha1jabb2gaTjabb6gaUjabbohaZbqaaiabbIcaOiabbMgaPjabb6gaUjabbogaJjabbYgaSjabbwha1jabbsgaKjabbMgaPjabb6gaUjabbEgaNjabbccaGiabbogaJjabb+gaVjabbYgaSjabbwha1jabb2gaTjabb6gaUjabbohaZjabbccaGiabbEha3jabbMgaPjabbsha0jabbIgaOjabbccaGiabbEgaNjabbggaHjabbchaWjabbohaZjabbMcaPaaaaiaaxMaacaWLjaGaei4waSLaeeyzauMaeeyCaeNaeiOla4IaeeiiaaIaee4naCJaeeyxa0faaa@C8CB@

### Construction of test set for benchmark of Dynalign z score classification method on known ncRNA sequence pairs

The sequence pair test set was constructed by randomly drawing and pairing real sequences from a pool of 309 known 5S rRNAs from the 5S ribosomal RNA database [48,76] and 482 known tRNAs from the Sprinzl database [48,77] (two tRNAs were not allowed because they contained an "X" (unknown) nucleotide that did not permit a dinucleotide shuffle to be done). This resulted in 755 real 5S rRNA and 896 real tRNA sequence pairs, whose distribution of percent identities was consistent with the distribution of percent identities of every possible pairwise alignment of the pools of all 5S rRNA and tRNA.

To test specificity, for each real sequence pair, a negative sequence pair was created by globally aligning the real pair, randomly shuffling the alignment columns (without regard for gap placement or local conservation), then removing the gaps. Prior to input to RNAz and QRNA, the shuffled sequences were globally re-aligned. The resulting test set contained 3,302 sequence pairs total.

It should be noted that two other methods for generating negative sequence pairs from real sequence pairs were additionally tried. The first was the "sre_shuffle" command line option in QRNA, which shuffles columns in an alignment while preserving gap position. Columns in the alignment are separated into three categories: nucleotide aligned to nucleotide, gap in sequence 1 aligned to nucleotide in sequence 2, and gap in sequence 2 aligned to a nucleotide in sequence 1; each column is shuffled only with other columns in its category. The second was the "SHUFFLEALN.PL" program [44] by Washietl *et al*, which, in the case of a pairwise sequence alignment as input, preserves gap position in the same fashion, but also preserves local conservation. Just as in QRNA's "sre_shuffle," alignment columns are divided into categories and each column is only shuffled with other columns in its category, but the nucleotide-aligned-to-nucleotide category is further subdivided into two categories – columns where the nucleotides are the same, i.e. conserved, and columns where nucleotides are different. However, benchmarks of the Dynalign *z *score classification method, RNAz, and QRNA showed that specificity for each program was sufficiently similar regardless of the method for generating negatives (data not shown), so for all tests a columnwise shuffle of a global alignment (without regard for gap placement or local conservation) was used to generate negative sequence pairs from real sequence pairs.

### Generation of controls for *z *score determination

Three methods were used for generating control sets for sequence pairs (only the Altschul-Erikson shuffle is used for generating control sets for single sequences):

(1) A columnwise shuffle (without regard for gap placement or local conservation) of a global alignment of the original sequence pair.

(2) Separately generating each sequence in the control pair by sampling from a first-order Markov chain as described in [41] without regard for alignment; for each sequence in the original pair, the nucleotide and dinucleotide frequencies are calculated, the first nucleotide in the control sequence is selected by sampling from the nucleotide frequencies of the original, then for the remainder of the sequence, each following nucleotide is sampled from the dinucleotide frequencies of the original, given that the first nucleotide is known. The dinucleotide frequencies of a sequence generated by this method would approach the dinucleotide frequencies of the original sequence in the limit of infinite length; however, since the lengths must be finite, the dinucleotide frequencies of the control are only approximately similar to the original sequence.

(3) The Altschul-Erikson dinucleotide shuffle [65] (implemented in Python by P. Clote, [78]) of each sequence in the original pair separately, which exactly preserves their nucleotide and dinucleotide frequencies, except that the shuffled sequence has the same first and last nucleotide as the original sequence.

All ΔG°_total_s in these trials were computed using the "global alignment" mode of Dynalign for both the input sequence pairs and the controls.

### Construction of ROC curves

To construct ROC curves for the Dynalign *z *score classification method, the *z *score cutoff was incremented from -11 to 3 in steps of 0.01 to generate test set sensitivity/specificity pairs ranging from 100% specificity to 100% sensitivity, then sensitivity was plotted as a function of the false positive rate (1 – specificity). Where the SVM probability (*P *value) was used as the classification cutoff, whether for RNAz or the Dynalign/LIBSVM classifier, the same was done, except *P *was incremented from 0 to 1 in steps of 0.001.

### Testing of QRNA and RNAz

QRNA (version 2.0.2c) [59] and RNAz (version 0.1.1) [43] require a pre-aligned sequence pair as input. RNAz can take a multiple sequence alignment as input, but here was only tested on sequence pairs. For benchmarks on test sets of known ncRNAs and negatives, this input was prepared by performing a global alignment of the two sequences. Whenever negative sequence pairs are produced from real sequence pairs by a columnwise shuffle of a global alignment, gaps are removed after the shuffle, and the sequences are globally re-aligned again to mimic the alignment that would be expected if these sequences were to appear in an actual ncRNA screen.

For whole genome screen tests, the genomic alignment windows used for input to the two programs were taken directly from the MUMmer or WuBLASTn alignment.

### Training and testing of the Dynalign/LIBSVM classifier

The LIBSVM [69] implementation of a support vector machine was employed for the Dynalign/SVM classifier method. The binary classifier SVM with a radial basis function (RBF) kernel was used. All LIBSVM classifier models were trained with command line parameters *-b 1 -c 32 -w-1 5 -g 6.10352e-05 *(the values were empirically determined), where *-b 1 *indicates that the model is trained to calculate probabilities of binary classification, *-c *specifies the value (C = 2^5^) of the penalty parameter of the error term, *-w-1 5 *specifies that the penalty of misclassifying negative sequence pairs as real sequence pair (i.e. misclassifying those labelled "-1" as those labelled "1") is 5 times the penalty specified by *-c *(this has the effect of reducing false positives), and *-g *specifies the value of γ in the RBF (γ = 2^-14^). Classification was done with the -*b 1 *parameter to output probabilities (*P *values), allowing for variation of the cutoff *P *value for classification and for construction of ROC curves. Input to LIBSVM was the Dynalign-computed ΔG°_total_, length of shorter sequence of the sequence pair, A, U, and C frequencies of sequence 1, and A, U, and C frequencies of sequence 2. Prior to input to LIBSVM, values for each parameter were scaled to the range [-1, 1]. The ranges for each parameter across the datasets is follows: ΔG°_total _was from -1868 to 0 (units of 10*kcal/mol); length of shorter sequence was from 50 to 150 nucleotides; frequencies of A, U, and C in sequence 1 were from 0.0701754 to 0.518519, from 0.0701754 to 0.518519, and from 0.0638298 to 0.42953, respectively; frequencies of A, U, and C in sequence 2 were from 0.0588235 to 0.623377, from 0.0588235 to 0.623377, and from 0.0402685 to 0.436364, respectively.

To train the SVM classifier, a training set containing every possible sequence pairing (not including pairing of sequences to themselves) was prepared from a pool of 309 known 5S rRNAs [48,76] and 479 known tRNAs [48,77] (two tRNAs that contained an "X" nucleotide and three tRNAs with three-way multibranch loops instead of the canonical four-way multibranch loops were removed from the Sprinzl database pool prior to this). This resulted in 47,586 5S rRNA and 114,481 tRNA sequence pairs. Two negative sequence pairs for each real sequence pair were generated: one by columnwise shuffle of a global sequence alignment, one by Altschul-Erikson shuffle of each sequence separately. This was intended to reduce the false positive rate by training the SVM classifier on a more diverse set of negative sequence pairs. The Dynalign ΔG°_total _(using the "local alignment" Dynalign mode and parameter *M *= 8) and the other SVM input data were computed for every sequence pair in the resulting training set of 486,201 data points. The free energies and sequence characteristics for each pair in the entire set are provided as Additional File 6 in the "Additional Files" section for reference, formatted for input to LIBSVM.

However, this set was unnecessarily large and biased towards tRNA, so for final SVM model training, only every 5th 5S rRNA and every 12th tRNA sequence pair were kept, producing a training set of a more realistic size of 9,517 5S rRNA and 9,540 tRNA sequence pairs, with 19,034 and 19,080 negative sequence pairs generated from them, for a training set size of 57,171 data points. All of the remaining 5S rRNA and one-half (every 2nd sequence pair, taken so that tRNA would not be over-represented) of the remaining tRNA sequence pairs were used to construct a test set for benchmarks of the Dynalign/LIBSVM classifier, RNAz, and QRNA.

Moreover, an additional 2,364 data points were added to the training set, which were calculated from alignments of sequences in the pool of 309 5S rRNA and 479 tRNA to themselves, with two negative sequence pairs generated from each real sequence pair as before (i.e. all of these 2,364 data points had 100% identity). This addition to the training set was done in order to train the Dynalign/LIBSVM classifier to more accurately classify high-identity genomic windows, of which there was a very large number in the whole genome screen (e.g. 21% of the genomic windows in the MUMmer whole genome screen method have identity above 98%, which does not reflect the distribution of percent identities in the original training set). Thus, the final training set size was 59,535 data points.

The result of the training is a LIBSVM model file that used by LIBSVM for classification and probability estimation. The model file is supplied as Additional File 7, although it should be noted that it will only work correctly on data scaled as described above.

### Sources of genomic data

All whole genome screens were conducted using the complete 4,639,675-nucleotide genome of *Escherichia coli *K-12 MG1655 [RefSeq NC_000913] and the complete 4,809,037-nucleotide main chromosome of *Salmonella enterica serovar Typhi *(*Salmonella typhi*) strain CT18 [RefSeq NC_003198].

For *E. coli*, the lists and genomic coordinates of 4,237 known open reading frames (ORFs) and 156 known ncRNAs were obtained from the NCBI Entrez Genome Project database [79]. The intergenic region size is 587,347 nucleotides.

For *S. typhi*, the lists and genomic coordinates of 4,594 known ORFs and 110 known ncRNAs were obtained from The Wellcome Trust Sanger Institute *S. typhi *database [80]. The intergenic region size is 604,213 nucleotides.

### Intergenic region alignment with WuBLASTn

To prepare genomic windows for a ncRNA screen of *E. coli *and *S. typhi *using WuBLASTn, intergenic regions of *E. coli *were constructed by taking the entire genome and removing all nucleotides in the 4,237 known ORFs, resulting in 587,347 nucleotides total. Each resulting segment was used as a WuBLASTn (version 2.0 [70], using default parameters) query against the entire *S. typhi *main chromosome. To maximize coverage, none of the resulting alignment blocks were filtered, except to throw out all those where the block length was less than 50 alignment columns. Alignment blocks length 50 to 150 (inclusive) were used as genomic windows directly; blocks with length greater than 150 were scanned with windows of size 150 alignment columns, step size 75. This resulted in 45,202 total genomic windows for the WuBLASTn ncRNA genomic screen. Reverse complements of sequences in each window were also scanned, resulting in 90,404 windows total as input to the Dynalign/LIBSVM classifier, RNAz, and QRNA, containing 10,265,161 alignment columns.

### Whole genome alignment with MUMmer

To prepare genomic windows for a ncRNA screen of *E. coli *and *S. typhi *using MUMmer, a whole genome alignment was generated using MUMmer 3.15 [71] with parameters *-b 1600 -c 10 *to increase genomic coverage (all other parameters were left at default values). All alignment columns containing nucleotides in known ORFs of either genome were removed from the resulting alignment blocks. The resulting alignment blocks were scanned with windows of size 150 alignment columns, step size 75, to generate windows for the genomic screen; because unlike WuBLASTn, the MUMmer whole genome alignment contains long stretches of gaps in some regions, some windows had to be dropped because one sequence in the window was aligned to only gaps for the other sequence. Additionally, windows containing a sequence less than 50 nucleotides in length were also dropped. After taking the reverse complement of each window, a total of 15,214 windows were input to the Dynalign/LIBSVM classifier, RNAz, and QRNA, containing 2,216,188 alignment columns.

## Availability and requirements

• **Project name: **Dynalign

• **Project home page: **

• **Operating system(s): **Platform independent

• **Programming language: **C++

• **Other requirements: **none

• **License: **GNU GPL

• **Any restrictions to use by non-academics: **none

## Abbreviations

5S rRNA – 5S subunit ribosomal RNA

AE – the Altschul-Erikson dinucleotide sequence shuffle method

columnwise – the columnwise sequence pair shuffle method

dinuc – the sampling from first-order Markov chain sequence shuffle method

ncRNA – non-coding RNA

ORF – open reading frame

PPV – positive predictive value

RBF – radial basis function

ROC – receiver operating characteristic

SCI – structure conservation index

SVM – support vector machine

tRNA – transport RNA

## Authors' contributions

AVU performed the computational experiments, analyzed the data, and drafted the manuscript. JMK optimized the Dynalign code to decrease runtime. DHM programmed the changes to Dynalign, conceived of the study, and contributed to the manuscript. All authors have read and approved the final manuscript.

## Supplementary Material

Additional File 1 Complete ROC curves for classification of sequence pairs by the Dynalign *z *score method. Adobe Acrobat PDF (version 4.0 or above) file showing complete ROC curves comparing effectiveness of Dynalign *z *score classification of sequence pairs using three control generation methods and two *M *parameter values (*M *= 6 and *M *= 8). This is the same sequence test set that Figures 3, 4 and 5 are based on. In all cases, increasing the value of the *M *parameter improves prediction quality. Dark and light green: controls generated by first-order Markov chain sampling, tests run using *M *= 6 and *M *= 8, respectively. Brown and orange: controls generated by Altschul-Erikson dinucleotide shuffle, tests run using *M *= 6 and *M *= 8, respectively. Dark and light blue: controls generated by the columnwise shuffle, tests run using *M *= 6 and *M *= 8, respectively.
Click here for file

Additional File 2 Side-by-side comparison of Dynalign, RNAz, and QRNA classifications for each window in the MUMmer whole genome screen. Plain text, whitespace-delimited tabular data file. Each row is a window in the MUMmer whole genome alignment (15,214 windows total) of *E. coli *and *S. typhi*. Columns 1, 2, and 3: *E. coli *start and end nucleotide indices and strand (plus or minus) for that window. Columns 4, 5, and 6: *S. typhi *start and end nucleotide indices and strand (plus or minus) for that window. Column 7: Dynalign/LIBSVM probability that the window is ncRNA. Column 8: RNAz probability that the window is ncRNA. Column 9: QRNA classification of the window (ncRNA, ORF, or other). Click here for file

Additional File 3 Side-by-side comparison of Dynalign, RNAz, and QRNA classifications for each window in the WuBLASTn whole genome screen. Plain text, whitespace-delimited tabular data file. Each row is a window in the WuBLASTn whole genome alignment (90,404 windows total) of *E. coli *and *S. typhi*. Columns 1, 2, and 3: *E. coli *start and end nucleotide indices and strand (plus or minus) for that window. Columns 4, 5, and 6: *S. typhi *start and end nucleotide indices and strand (plus or minus) for that window. Column 7: Dynalign/LIBSVM probability that the window is ncRNA. Column 8: RNAz probability that the window is ncRNA. Column 9: QRNA classification of the window (ncRNA, ORF, or other). Click here for file

Additional File 4 MUMmer whole genome screen input data to the Dynalign/LIBSVM classifier. Plain text data file formatted for input to LIBSVM (not scaled). This is the MUMmer whole genome screen dataset input to the Dynalign/LIBSVM classifier (before scaling). There is a one-to-one correspondence between rows of this file and rows of Additional File 2 – that is, row *N *in this file corresponds to the window described in row *N *in Additional File 2. Column 1 is the data label (all windows are initially assumed negatives and labelled "-1," but this is irrelevant for these purposes as this is essentially just a placeholder column for LIBSVM). Column 2 is the Dynalign-computed ΔG°_total_; column 3 is the length of shorter sequence; columns 4, 5, and 6 are A, U, and C frequencies of sequence 1 (*E. coli*); columns 7, 8, and 9 are A, U, and C frequencies of sequence 2 (*S. typhi*). Click here for file

Additional File 5 WuBLASTn whole genome screen input data to the Dynalign/LIBSVM classifier. Plain text data file formatted for input to LIBSVM (not scaled). This is the WuBLASTn whole genome screen dataset input to the Dynalign/LIBSVM classifier (before scaling). There is a one-to-one correspondence between rows of this file and rows of Additional File 3 – that is, row *N *in this file corresponds to the window described in row *N *in Additional File 3. Column 1 is the data label (all windows are initially assumed negatives and labelled "-1," but this is irrelevant for these purposes as this is essentially just a placeholder column for LIBSVM). Column 2 is the Dynalign-computed ΔG°_total_; column 3 is the length of shorter sequence; columns 4, 5, and 6 are A, U, and C frequencies of sequence 1 (*E. coli*); columns 7, 8, and 9 are A, U, and C frequencies of sequence 2 (*S. typhi*). Click here for file

Additional File 6 LIBSVM datasets for every possible sequence pair of 5S rRNA, tRNA, and negative sequences. Nine plain text data files formatted for input to LIBSVM (not scaled) and three plain text files containing sequence codes for the LIBSVM files, all archived with GNU 'tar' and compressed with GNU 'gzip'. Our training and testing sets for the Dynalign/LIBSVM classifier were prepared from this dataset as described in "Methods." The file 'LIBSVM-set.5s-real' is every possible pairing of known 309 5S rRNA sequences in our database, not counting sequences paired with themselves. The file 'LIBSVM-set.trna-real' is every possible pairing of known 479 tRNA sequences in our database, not counting sequences paired with themselves. The file 'LIBSVM-set.100ident-real' is the 309 5S rRNA and 479 tRNA sequences paired with themselves (i.e. real sequence pairs of 100% identity). The files denoted 'neg-column' are columnwise-shuffled negatives generated from the corresponding real sequences; the files denoted 'neg-AE' are negatives generated from the corresponding real sequences by the Altschul-Erikson shuffle (see "Methods" for description of both shuffles). The files denoted 'seqlist' contain the codes for sequence pairs (or for sequences aligned with themselves) with lines in a one-to-one correspondence with the appropriate LIBSVM files – for example, line 42 of file 'seqlist.5s-pairs' contains the codes of the two 5S rRNA sequences which were used to generate the data on lines 42 in files 'LIBSVM-set.5s-real', 'LIBSVM-set.5s-neg-column', and 'LIBSVM-set.5s-neg-AE'. For LIBSVM files, column 1 the data label (1 for real, -1 for negative); column 2 is the Dynalign-computed ΔG°_total_; column 3 is the length of shorter sequence; columns 4, 5, and 6 are A, U, and C frequencies of sequence 1; columns 7, 8, and 9 are A, U, and C frequencies of sequence 2. Click here for file

Additional File 7 LIBSVM model file for the Dynalign/LIBSVM classifier. The model file for a LIBSVM classifier, trained as described in "Methods." LIBSVM classifications with this model file also outputs a probability of prediction (*P *value), in addition to the prediction itself. Use this with LIBSVM on datasets that have been scaled as described in "Methods" and note that datasets scaled differently will be incorrectly classified. The input dataset should be a plain text, whitespace-delimited tabular file formatted as described in Additional File 6 and in the LIBSVM documentation [69]. Click here for file

## Figures and Tables

**Table 1:** Percent of known base pairs in predicted suboptimal structures for single sequences.

	**Maximum percent change in free energy from lowest free energy structure**					
**RNA Type^1^**	**1%**	**5%**	**10%**	**20%**	**30%**	**50%**

SSU (16S) rRNA	74.5 ± 21.9 (80.5 ± 16.0)^2^	88.1 ± 14.9 (96.8 ± 2.7)^2^	97.1 ± 13.6 (97.2 ± 1.4)^2^	99.2 ± 8.2 (97.2 ± 1.4)^2^	99.3 ± 7.2 (97.2 ± 1.4)^2^	99.3 ± 3.4 (97.2 ± 1.4)^2^
LSU (23S) rRNA	84.4 ± 8.9 (91.9 ± 13.4)^2^	96.8 ± 3.8 (97.9 ± 1.2)^2^	98.1 ± 1.2 (98.0 ± 0.7)^2^	98.1 ± 1.2 (98.0 ± 0.7)^2^	98.1 ± 1.2 (98.0 ± 0.7)^2^	98.1 ± 1.2 (98.0 ± 0.7)^2^
5S rRNA	74.5 ± 25.6	88.1 ± 20.1	97.1 ± 7.4	99.2 ± 1.6	99.3 ± 1.4	99.3 ± 1.4
Group I Intron	79.0 ± 12.6	93.5 ± 7.6	98.3 ± 1.4	98.4 ± 1.4	98.4 ± 1.4	98.4 ± 1.4
Group I Intron – 2	74.4 ± 13.6	92.8 ± 8.0	97.1 ± 1.7	97.1 ± 1.7	97.1 ± 1.7	97.1 ± 1.7
Group II Intron	91.9 ± 5.7	100.0 ± 0.0	100.0 ± 0.0	100.0 ± 0.0	100.0 ± 0.0	100.0 ± 0.0
RNase P	79.8 ± 11.0	95.5 ± 2.6	98.4 ± 1.2	98.4 ± 1.2	98.4 ± 1.2	98.4 ± 1.2
RNase P – 2	75.0 ± 7.9	95.6 ± 4.9	98.3 ± 1.5	98.3 ± 1.5	98.3 ± 1.5	98.3 ± 1.5
SRP RNA	73.1 ± 25.2	90.7 ± 14.3	95.5 ± 8.2	97.1 ± 2.7	97.2 ± 2.6	97.2 ± 2.6
tRNA	87.0 ± 18.2	94.5 ± 13.3	97.9 ± 7.8	99.3 ± 4.6	99.6 ± 3.2	99.8 ± 1.0
**Average^3^**	**80.5 ± 6.7**	**93.4 ± 4.2**	**97.8 ± 1.3**	**98.7 ± 0.9**	**98.8 ± 0.9**	**98.8 ± 1.0**

^1^The database of structures was assembled for previous studies of secondary structure prediction [48] and is derived from a diverse set of databases [76, 77, 81-84].

^2^The large and small subunit rRNA sequences are divided into domains of less than 700 nucleotides for structure prediction. In parenthesis are the accuracies when the whole sequence is folded at once.

^3^The average is calculated excluding the second database of Group II introns and RNase P sequences as was done in [48].

The percent of known base pairs contained in at least one predicted suboptimal structure within a specified percent difference in free energy from the minimum free energy. For example, 99.2% of known base pairs in 5S rRNA secondary structures are found on average within the predicted structures with less than 20% difference in free energy from the lowest free energy structure. These numbers were calculated from an energy dot plot using the thermodynamic parameters of [49]. On average, 98.7% of known base pairs occur in at least one suboptimal secondary structure within 20% or less difference in free energy from the minimum free energy structure. This accuracy remains similar as the percent energy difference is increased to 30% or 50%.

**Table 2:** Secondary structure prediction accuracy with and without speed improvements for tRNA and 5S rRNA sequences.

	**Without speed improvements**			**With speed improvements**		
**RNA type**	**Sensitivity**	**PPV**	**Best sensitivity**	**Sensitivity**	**PPV**	**Best sensitivity**

tRNA	92.9 ± 12.6	92.7 ± 14.3	98.8 ± 4.3	93.0 ± 12.3	92.7 ± 14.1	99.1 ± 2.8
5S rRNA	91.7 ± 7.0	82.0 ± 7.1	97.9 ± 3.2	91.7 ± 6.9	81.9 ± 7.0	98.0 ± 3.0

Sensitivity is the percent of known base pairs correctly predicted. Positive predictive value (PPV) is the percent of predicted base pairs that are in the known structure. Best sensitivity is the sensitivity of the most sensitive structure in a set of 750 suboptimal structures, i.e. structures with folding free energy change similar to the lowest free energy structure. Sensitivity and positive predictive value are calculated as described previously [46]. The tRNA and 5S rRNA datasets are sets of randomly chosen sequences, set sizes 40 and 14, respectively, used for benchmarks previously [46]. Accuracies are reported as averages for all pairwise combinations of sequences, and single standard deviations are reported as errors. The average accuracy of secondary structure prediction by Dynalign is essentially unchanged by pre-filtering base pairs and changing the implementation of the *M *parameter. Without speed improvements, *M *was set to 15 for both tRNA and 5S rRNA. With speed improvements, *M *was 6 for tRNA and 7 for 5S rRNA for these benchmarks.

**Table 3:** Dynalign calculation time and memory requirements.

				**Dynalign before acceleration**			**Dynalign after acceleration**		
**System**	**Sequence 1**	**Sequence 2**	**Length (nt)**	***M***	**Time (hr:min)**	**Memory (MB)**	***M***	**Time (hr:min)**	**Memory (MB)**

tRNA	RD0260	RE6781	77	15	0:22 (0:24)	33 (57)	6	0:01 (0:01)	12 (24)
5S rRNA	*H. volcanii*	*A. globiformis*	122	15	1:11 (1:09)	76 (85)	6	0:03 (0:03)	21 (30)
R2 3' UTR RNA	*D. takahashii*	*D. melanogaster*	217	24	26:05	491	8	0:39 (0:35)	81 (104)

Calculation times and memory use are reported for a 3.2 GHz Intel Pentium 4 with 1 GB RAM running Red Hat Enterprise Linux using the gcc 3.2.3-42 compiler. In parentheses are time and memory requirements on a laptop with a 3.06 GHz Pentium 4 processor and 1 GB of RAM running Microsoft Windows XP Professional using the Microsoft C++ .NET 2002 compiler. For Linux, CPU time is reported; for Windows, wall time is reported. "Length" is length of the first sequence. Sequences are obtained from [76, 77, 85, 86]. Note that this is the time requirement including suboptimal secondary structure prediction. Slightly less than half the computer time is required to find only the lowest free energy common structure.

**Table 4:** Sensitivities of the Dynalign/LIBSVM classifier, RNAz, and QRNA broken down by percent identity.

		**% sensitivity**					**% specificity**				
**% identity range**		**N**	**% of real set**	**Dynalign (*P *> 0.819)**	**RNAz (*P *> 0.789)**	**QRNA**	**N**	**% of negative set**	**Dynalign (*P *<= 0.819)**	**RNAz (*P *<= 0.789)**	**QRNA**

[0	10)	0	0	N/A	N/A	N/A	0	0	N/A	N/A	N/A
[10	20)	0	0	N/A	N/A	N/A	0	0	N/A	N/A	N/A
[20	30)	3	0.0033	100.0	0.0	66.6667	49	0.0271	97.9592	100.0	95.9184
[30	40)	1337	1.4767	71.4286	34.6298	48.6163	9037	4.9907	99.1922	97.0233	97.8975
[40	50)	22328	24.6612	63.6465	63.1539	46.5559	85008	46.9455	99.3612	98.8542	99.2789
[50	60)	42733	47.1984	73.0606	75.6488	56.2469	55602	30.7061	99.2320	99.5018	99.0306
[60	70)	17346	19.1586	88.4930	92.2461	86.0775	23952	13.2274	99.1566	99.3028	98.8143
[70	80)	4061	4.4854	94.5826	94.5087	94.1394	4672	2.5801	97.5813	98.4375	97.9238
[80	90)	2035	2.2477	95.5774	91.9410	93.9066	2062	1.1387	90.8341	98.1086	96.6052
[90	100)	654	0.7223	98.4709	72.6300	59.7859	654	0.3612	60.5505	96.7890	95.2599
[100 ]		42	0.0464	92.8571	61.9048	11.9048	42	0.0232	61.9048	90.4762	95.2381
**totals**		**90539**	**100.0**	**75.3366**	**81.2854**	**62.0108**	**181078**	**100.0**	**98.9938**	**98.9927**	**98.9905**

A comparison of sensitivities of the three ncRNA classification/detection programs within each percent identity range. *N *is the number of sequences within each range. Probability cutoffs for RNAz and the Dynalign/LIBSVM classifier were selected such that overall specificities for the entire test set match the specificity of QRNA as closely as possible.

**Table 5:** Detection of long ncRNAs using scanning windows.

	**percent identity of entire alignment**	**total number of scanning windows**	**number of scanning windows with *P *> 0.5**	**number of scanning windows with *P *> 0.9**	**number of scanning windows with *P *> 0.99**
**16S rRNA**					

*Borrelia burgdorferi *and *Bacillus subtilis*	74.5%	30	17	12	6
*Homo sapiens *(mitochondrial) and *Thermotoga maritima*	39.3%	30	1	1	0
*Archaeoglobus fulgidus *and *Borrelia burgdorferi*	61.8%	30	22	17	14
**23S rRNA**					

*Escherichia coli *and *Thermoplasma acidophilum*	59.4%	57	49	41	37
*Bacillus subtilis *and *Bos taurus*	37.1%	57	35	26	21
*Bacillus subtilis *and *Thermoproteus tenax*	60.1%	61	3	2	1

The Dynalign/LIBSVM classifier is used to compute *P *values for sets of 150-nucleotide scanning windows iterating (in steps of 75 nucleotides) through global alignments of three 16S and three 23S rRNA pairs randomly selected from a database [48]. The quantity of windows above three *P *value cutoffs is listed, indicating that long ncRNAs can be detected with short scanning windows.

**Table 6:** Comparison of three ncRNA detection programs on a whole genome screen using the MUMmer alignment.

	**probability cutoff for ncRNA classification**						
		
	***P *> 0.5**		***P *> 0.9**		***P *> 0.99**		
	
	**Dynalign**	**RNAz**	**Dynalign**	**RNAz**	**Dynalign**	**RNAz**	**QRNA**
**Known ncRNAs found (percent of total known ncRNAs in parentheses)**							
*E. coli *(156 ncRNAs known)	128 (82.05)	125 (80.13)	123 (78.85)	104 (66.67)	107 (68.59)	91 (58.33)	67 (42.95)
*S. typhi *(110 ncRNAs known)	103 (93.64)	98 (89.09)	102 (92.73)	84 (76.36)	93 (84.55)	70 (63.64)	64 (58.18)
**Number of contiguous, non-overlapping hits that are not known ncRNAs (i.e. novel ncRNA candidates)**							
*E. coli*	1,183	1,255	872	996	578	678	661
*S. typhi*	1,178	1,255	857	977	568	662	634
**Number of nucleotides classified as ncRNA that are not in known ncRNAs (i.e. nucleotides in novel ncRNA candidates)**							
*E. coli *(each strand = 4,639,675 nt)	169,580	174,790	123,563	128,343	81,936	80,054	87,577
*S. typhi *(each strand = 4,809,037 nt)	163,037	174,126	117,277	126,393	76,289	79,713	88,099
**Total number of nucleotides classified as ncRNA (i.e. nucleotides in both known and unknown ncRNAs)**							
*E. coli *(each strand = 4,639,675 nt)	224,051	222,817	175,174	166,676	129,086	104,428	113,090
*S. typhi *(each strand = 4,809,037 nt)	213,549	218,867	166,187	162,077	122,269	102,464	114,434

QRNA, RNAz, and the Dynalign/LIBSVM classifier are compared in their ability to detect known ncRNA in the *E. coli *and *S. typhi *genomes, based on a MUMmer whole genome alignment. For RNAz and the Dynalign/LIBSVM classifier, results are listed for three P value classification cutoffs. "Number of nucleotides" = number of nucleotides on the plus strand + number of nucleotides on the minus strand, not accounting for overlap of complementary strands.

**Table 7:** Comparison of three ncRNA detection programs on a whole genome screen using WuBLASTn genomic windows.

	**probability cutoff for ncRNA classification**						
		
	***P *> 0.5**		***P *> 0.9**		***P *> 0.99**		
	
	**Dynalign**	**RNAz**	**Dynalign**	**RNAz**	**Dynalign**	**RNAz**	**QRNA**
**Known ncRNAs found (percent of total known ncRNAs in parentheses)**							
*E. coli *(156 ncRNAs known)	147 (94.23)	148 (94.87)	141 (90.38)	140 (89.74)	128 (82.05)	124 (79.49)	121 (77.56)
*S. typhi *(110 ncRNAs known)	109 (99.09)	108 (98.18)	107 (97.27)	106 (96.36)	103 (93.64)	95 (86.36)	100 (90.91)
**Number of contiguous, non-overlapping hits that are not known ncRNAs (i.e. novel ncRNA candidates)**							
*E. coli*	2,569	1,898	1,828	1,568	1,211	1,257	1,403
*S. typhi*	3,936	2,503	2,440	1,986	1,520	1,520	1,611
**Number of nucleotides classified as ncRNA that are not in known ncRNAs (i.e. nucleotides in novel ncRNA candidates)**							
*E. coli *(each strand = 4,639,675 nt)	324,033	275,704	235,227	221,802	167,157	174,682	206,840
*S. typhi *(each strand = 4,809,037 nt)	514,650	387,099	352,039	296,608	235,018	220,485	248,724
**Total number of nucleotides classified as ncRNA (i.e. nucleotides in both known and unknown ncRNAs)**							
*E. coli *(each strand = 4,639,675 nt)	385,611	332,000	292,940	270,267	220,352	209,807	242,534
*S. typhi *(each strand = 4,809,037 nt)	570,994	436,137	405,022	338,254	283,999	250,610	283,508

QRNA, RNAz, and the Dynalign/LIBSVM classifier are compared in their ability to detect known ncRNA in the *E. coli *and *S. typhi *genomes, based on genomic scanning windows prepared using WuBLASTn. For RNAz and the Dynalign/LIBSVM classifier, results are listed for three *P *value classification cutoffs. "Number of nucleotides" = number of nucleotides on the plus strand + number of nucleotides on the minus strand, not accounting for overlap of complementary strands.
