# Supplementary figures and images for: Detection of non-coding RNAs on the basis of predicted secondary structure formation free energy change
Source: BMC Bioinformatics. 2006 Mar 27;7:173. doi: 10.1186/1471-2105-7-173 (PMC1570369; doi:10.1186/1471-2105-7-173)

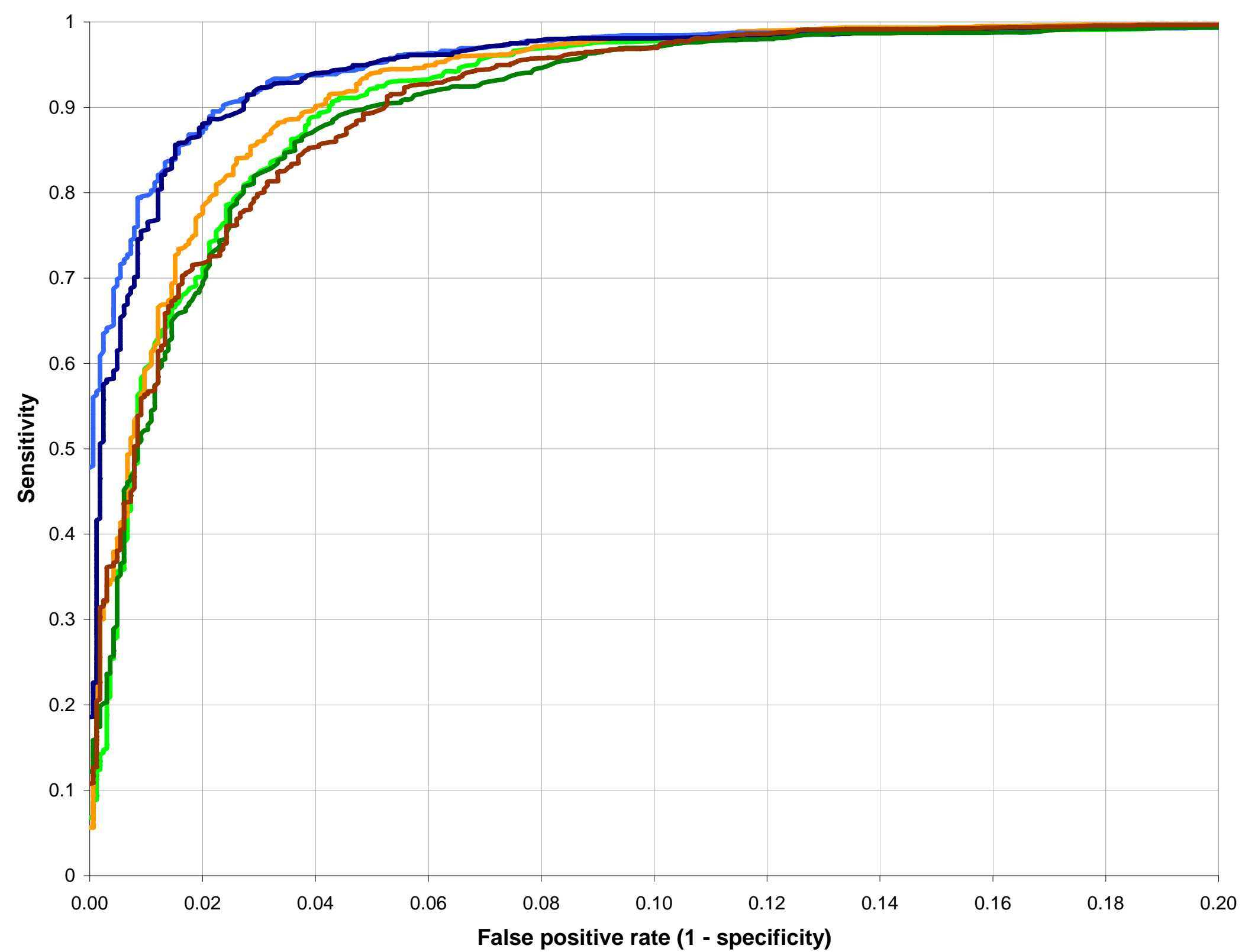

Supplement: Additional File 1 — Complete ROC curves for classification of sequence pairs by the Dynalign z score method. Adobe Acrobat PDF (version 4.0 or above) file showing complete ROC curves comparing effectiveness of Dynalign z score classification of sequence pairs using three control generation methods and two M parameter values (M = 6 and M = 8). This is the same sequence test set that Figures 3, 4 and 5 are based on. In all cases, increasing the value of the M parameter improves prediction quality. Dark and light green: controls generated by first-order Markov chain sampling, tests run using M = 6 and M = 8, respectively. Brown and orange: controls generated by Altschul-Erikson dinucleotide shuffle, tests run using M = 6 and M = 8, respectively. Dark and light blue: controls generated by the columnwise shuffle, tests run using M = 6 and M = 8, respectively. [file 1471-2105-7-173-S1.pdf]
